# The Effects of a Visual Stimuli Training Program on Reaction Time, Cognitive Function, and Fitness in Young Soccer Players

**DOI:** 10.3390/s22176680

**Published:** 2022-09-03

**Authors:** Georgia Theofilou, Ioannis Ladakis, Charikleia Mavroidi, Vasileios Kilintzis, Theodoros Mirachtsis, Ioanna Chouvarda, Evangelia Kouidi

**Affiliations:** 1 Laboratory of Sports Medicine, Department of Physical Education and Sports Sciences, Aristotle University of Thessaloniki (AUTh), P.C. 57001 Thessaloniki, Greece; 2 Laboratory of Computing, Medical Informatics and Biomedical—Imaging Technologies, School of Medicine, Aristotle University of Thessaloniki, P.C. 54124 Thessaloniki, Greece; 3 Ophthalmology Department, 424 Military Hospital, P.C. 56429 Thessaloniki, Greece

**Keywords:** reaction time, cognitive function, physical fitness, FITLIGHT Trainer, visual field, VR, soccer players

## Abstract

The purpose of the present study was to examine whether a visual stimuli program during soccer training can affect reaction time (RT), cognitive function, and physical fitness in adolescent soccer players. Thirty-eight male soccer players aged 10–15 were randomly assigned to either the intervention (Group A) or the control group (Group B). At baseline and at the end of the 6-month study FITLIGHT Trainer, the Cognitive Function Scanner Mobile Test Suite, a Virtual Reality (VR) game, and the ALPHA—Fitness and the Eurofit test batteries were used to measure participants’ abilities. After the baseline assessment, Group A followed their regular soccer training combined with a visual stimuli program, while Group B continued their regular soccer training program alone for 6 months. At the end of the 6-month study, Group A showed statistically significant improvements in simple RT by 11.8% (*p* = 0.002), repeated sprints by 13.4% (*p* ≤ 0.001), and Pen-to-Point Cognitive Function by 71.62% (*p* < 0.001) and 72.51% for dominant and non-dominant hands, respectively. However, a between-groups analysis showed that there was no statistically significant difference between the two groups in most of the measurements studied. In conclusion, a visual stimuli training program does not seem to add any value to the traditional soccer training program for adolescents. Nevertheless, this study helps to underline the potential of newly emerging technology as a tool for the assessment of RT.

## 1. Introduction

Sports vision is an emerging field of ophthalmology aiming to enhance both visual function and athletic performance. In the existing literature, various studies have been conducted that indicate the importance of vision in sports performance [1,2,3,4]. In their study, Spera et al. presented interesting results regarding the impact of vision on postural stability, conducting an experiment between sighted and visually impaired athletes [5].

Several studies have shown that vision plays a significant role in sports practice and, therefore, visual education should be a significant part of athletes’ exercise training [6,7]. Soccer players were found to have better peripheral vision, depth perception, and visual tracking of a moving object compared to non-athletes [8]. The two main metrics/variables of sports vision are visual reaction time and peripheral vision [1,9]. Visual reaction time is directly associated with movement control and regulation processes that are affected by the central nervous system (CNS) and muscular effects. Motor reaction time is quite different as it is defined as the time between the signal and the completion of an action, including both sensory and motor characteristics [1,10]. It is clear vision plays a critical role for athletes as they have to carefully attend to and process the information from various visual stimuli related to the very concept of the specific sport. Optimal central–peripheral simultaneity is vital in this way [1,11,12].

It has been noticed that reaction time (RT) plays an important role in many fields, such as academics, sports, and daily life tasks [13,14,15]. It is related to the sensorimotor cycle, the detection of the initial stimulus, the transfer via the afferent nerves, the generation of the response by CNS, and the final response [13,16,17]. There is a differentiation between simple RT (SRT) and complex RT (CRT). The SRT is the interval between stimulus appearance and the given response [13,18], while the CRT is the recognition and selection of a response to various stimuli [13,19].

Several factors can influence the RT, such as exhaustion, physical fitness, experience, motivation, gender, age, or dominance of the extremity with which one responds. There are also other factors that are related to the stimulus, such as its intensity or duration [13,18,20,21]. Other important internal factors are the cognitive process and attention, which are involved in the RT [13,22,23,24]. They affect the activation and selection processes, distribution, and maintenance of psychological activity [13,25] and show anatomical and functional complexity [14,26]. One of its types especially, selective attention, includes the ability to attend to specific stimuli and ignore others [13,26,27]. 

Because of the rapid technological evolution and the emergence of new Virtual Reality (VR) solutions, there is an increased research interest in the role of VR in the evaluation of the physical performance and rehabilitation in patients with Parkinson’s, imbalance, stroke, etc. [28,29,30]. VR can be an important tool in the quick assessment of physical performance.

For the evaluation of RT in sports, a few studies have used the FITLIGHT Trainer^TM^ (FITLIGHT Sports Corp., Canada). Specifically, Zwierko et al. showed that non-athletes had longer RTs compared to handball players [31], while Fischer et al. [32] reported the use of training and analysis of the RT in the United States Air Force [32]. Moreover, Zurek et al. investigated the simple and complex RTs of football players after knee surgery and a rehabilitation program [33].

The evaluation of the effectiveness of a visual training program in sports was investigated by Abernethy et al. [34]. Among the implemented stimuli were symbols, shapes, patterns, and colors. The stimuli were displayed in painted graphics or objects, and the participants were asked to respond with simple ocular adjustments combined with simple motor actions. Another study deployed Nike Vapor Strobe glasses for the assessment of sports training. The results indicated that the use of special training glasses enhanced central visual field motion sensitivity and transient attention abilities. However, there was no difference in their peripheral motion sensitivity and multiple-object tracking [35]. Although the aforementioned literature shows a strong relationship between RT and variables such as selective attention and physical condition, there is no study using VR technology for RT assessment and visual function analysis in young athletes.

Moreover, it has been observed that physical fitness has an impact on RT [13,36,37] by helping the development of cognitive function and different aspects of attention [13,38,39,40]. Moreover, some studies have shown that fit individuals are associated with quicker RTs [13,41,42]. Furthermore, it has been shown that cognitive processes and attention are involved in RT [13,22,23,24]. Therefore, the combination of physical exercise and the development of cognitive functioning could be an appropriate formula to improve RT not only in athletes but in the general population.

Thus, the aim of the study was to examine whether a visual stimuli program during soccer training can affect RT, cognitive function, and physical fitness in adolescent soccer players. Specifically, the research hypotheses of this study are:

1. There is a significant relationship between RT and physical performance, as well as cognitive function, in adolescent soccer players.
2. A visual stimuli training program can improve the RT, cognitive function, and physical performance of adolescent soccer players.

## 2. Materials and Methods

### 2.1. Study Population

This study is a randomized control trial that initially involved 42 male soccer players aged 10–15 without any ocular pathology. At the end of the 6-month duration of the study, 38 soccer players completed the study, 18 in the intervention group (Group A) and 20 in the control group (Group B). Their soccer experience was between 1 and 7 years.

### 2.2. Study Protocol

To carry out the research, a large, high-performance soccer academy in Northern Greece was contacted. The young soccer players and their parents were informed about the aim and methodology of the study. All baseline tests were performed within a day, in the morning hours, between 9:00 and 12:00 am. After a 5 min warm-up, the testing battery started with the repeated sprint test (5 min in total) and continued with the assessment of RT using FITLIGHT Trainer (10 min) and visual field VR test (up to 3 min). Thereafter, participants performed sit-and-reach (3 repetitions of 2 s, 1 s rest), a figure drawing test and pen-to-point test (both in seconds according to participant’s performance), hand grip test (3 repetitions of 2 s, 1 s rest), and manual dynamometry test (3 repetitions of 5 s, 10 s rest.), There was a 10 min rest between the different tests. The tests were clearly explained and shown. Practice trials were not provided. The tests were performed in different places of the sports facilities, and each participant was called separately.

After baseline assessment, volunteers were randomly divided into two groups using the program http://www.randomizer.org/ (accessed on 2 September 2021). Athletes of group A followed a 6-month soccer training program with visual stimuli 5 times per week, lasting 15 min each time, which preceded their regular soccer training program. Soccer players of Group B followed the standard soccer training program but without any visual stimuli. The soccer training protocols of the developmental age guide of the Hellenic Football Federation suggest an individual training program according to position (goalkeeper, defender, midfielder, and forwarder) for athletes aged 10–12 years old and a subgroup training program for athletes aged 13 to 15 years old. In this case, the athletes were divided into two subgroups playing against each other. The most common combination was one subgroup consisting of the goalkeeper, the defenders, and half of the midfielders and the other subgroup consisting of the other half of the midfielders and forwarders. The athletes had to attend at least the 85% of the exercise training sessions to be included in the analysis. After the 6-month period, the measurements were repeated in both groups. All assessors were blinded to the athlete’s group allocation. 

In addition, informed and written consent was obtained from parents or legal guardians of the soccer players. The required approval was obtained from the Ethics Committee of the Faculty of Physical Education and Sport Sciences of the Aristotle University of Thessaloniki (approval number 68/2021).

### 2.3. Measurements

#### 2.3.1. Reaction Time Assessment

The FITLIGHT Trainer (FITLIGHT Sports Corp., Aurora, ON, Canada) was used to measure the RT. This is a wireless system consisting of three sensors, which were placed in front of each athlete in a semicircular arrangement with a radius of 35 cm and a distance of 20 cm between them. Each unit was switched on and off when the trainee passed the lower extremity over it. Two measurements were performed on each athlete, and the simple RT (SRT) and complex RT (CRT) were calculated. In the first measurement, the athlete used the dominant lower extremity, and in the second, the athlete used the dominant lower extremity when the illuminated unit gave a green stimulus and the non-dominant one when given a red stimulus.

#### 2.3.2. Physical Fitness Assessment

To assess physical fitness, athletes were asked to perform selected tests from the European Physical Fitness Test Battery (EUROFIT) [43] and the Assessing Levels of Physical Activity (ALPHA) Health-Related Fitness Test Battery for Children and Adolescents [44]. Specifically, the following tests were carried out:

(a) **Manual dynamometry**

KForce Link (Kinvent Biomecanique, Montpellier, France; Sf = 75 Hz) pull dynamometer was used to assess the isometric mid-thigh pull forces. The dynamometer was linked to a chain with a bar end. Each athlete was asked to pull the bar as strongly as possible. The test was repeated three times. The dynamometer was wirelessly linked to the mobile app, where the results were stored. The value of interest for our measurements was RFD (rate of force development), which depicts the ability of a muscle to apply force very quickly and is directly related to performance.

(b) **Hand grip**

A digital hand grip dynamometer (Jamar Smart Hand Dynamometer, PATTERSON MEDICAL, Saint Paul, MN, USA) was used. Firstly, the athlete held the instrument using the dominant hand. The task was to grip it as strongly as possible without supporting the hand on the body. The test lasted 2 s and was repeated 3 times. Then, the procedure was repeated for the non-dominant hand. The best of the three efforts for each hand was written down.

(c) **Sit-and-reach test**

To assess flexibility, each athlete was asked to sit on the floor with legs stretched out straight ahead. The athlete was placing the soles against a wooden box with the knees pressed flat to the floor. Placing the hand on top of each other, the athlete was to bend forward along a measuring scale as far as possible. After some trials, the athlete bent forward and was instructed to keep that position for 1–2 seconds. During this period, the distance reached was recorded.

(d) **Repeated Sprint test**

To assess speed, agility, and coordination, 2 parallel lines at a 5 m distance were drawn, and each line was 1.2 m in length. We put cones at both ends of the 2 lines, and we asked each athlete to run as fast as possible to the other line and come back, with both feet behind the lines when reaching them, and repeat this 5 times.

### 2.4. Cognitive Tests

The assessment of the cognitive function was performed using a cognitive function test application (Cognitive Function Scanner Mobile Test Suite, Denmark), the figure drawing test, and the pen-to-point test to evaluate isuomotor functioning.

(a) **Figure Drawing Test**

Each athlete was instructed to take the pen (pencil) in the left/right hand (dominant hand). The task was to draw on top of the curved line as accurately as possible. The athlete began up from the top mark and drew all the way round. The direction was chosen by the athlete and was to have remained the same until the end of the measurement. Time was measured, but accuracy was the most important factor. There were not to be lifts of the pen. The hand of the initial measurement was the dominant hand. We repeated the procedure for the non-dominant hand.

(b) **The Pen-to-Point Test**

In this test, the athlete began with the dominant hand again. The task was to point to the center of each cross in this line (point out the line of crosses) with the tip of the digital pen (pencil). The test was repeated using the non-dominant hand.

### 2.5. Visual Field

**Figure 1:**
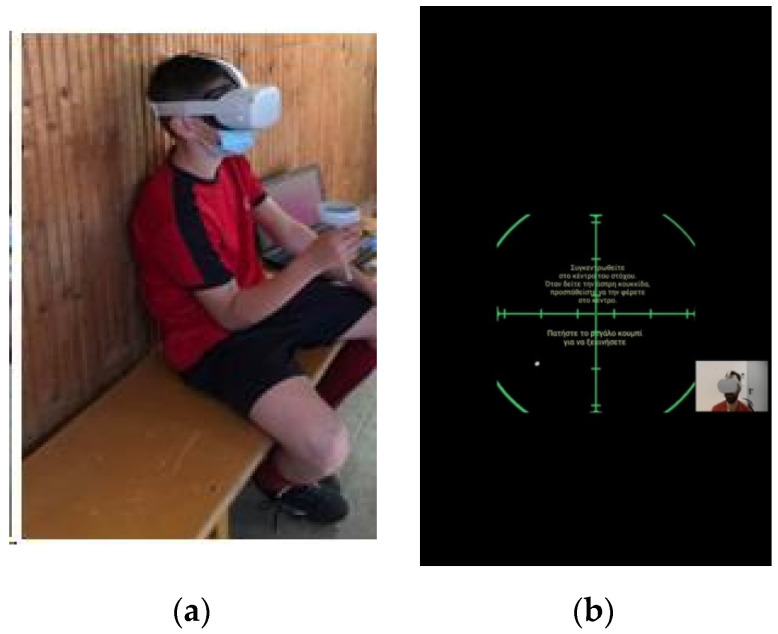
(**a**) VF assessment using VR equipment (Oculus GO—Oculus Quest 2). (**b**) The internal virtual environment of the application as seen by the athlete.

The assessment of the visual field was completed using an application and wearable VR equipment (Oculus GO—Oculus Quest 2) designed and programmed by the Laboratory of Computing, Medical Informatics and Biomedical—Imaging Technologies of Aristotle University of Thessaloniki, School of Medicine. The intention of the application is to miniaturize the VF test to be provided as an efficient tool for standard clinical practice. Each athlete watched a green cross and had to focus on its center, and an illuminating white ball appeared from different angles from the periphery (Figure 1). The athlete had to press the button of a remote control as fast as possible after the moment the ball appeared. The application internally calculates the different angles of appearance of the ball (angle φ in rad), the RT (s), extracts descriptive statistics regarding the calculated features and provides the final isopter of the examinee (unilateral and bilateral).

### 2.6. Visual Stimuli Soccer Training Program

After baseline assessment, athletes of Group A followed their regular soccer training combined with the visual stimuli program 5 times per week for 6 months. Each training session started with the visual stimuli training program, which lasted 15 min, and then continued with the regular soccer training program. The visual stimuli program included technical exercises (transfer, reception, driving, shooting), as well as exercises for strengthening, reflexes, and neuromuscular coordination using light sensors (Blazepod Flash Reflex Training System, Miami, FL, USA), a soccer ball, and training cones.

A random combination of 8 different exercises was followed for 15 min in each training session only for Group A. The exercises were the following: 

Exc. 1 (shooting): The athlete was in front of the goalpost, and when the illuminated sensor gave a green light, he had to shoot.

Exc. 2 (skipping): The athlete had to skip when the illuminated sensor gave a green light, and he had to stop when the sensor gave a red light.

Exc. 3 (shooting): The athlete had to shoot to the goalpost using the dominant leg when the illuminated sensor gave a green light and use the non-dominant leg when the illuminated sensor gave a red light.

Exc. 4 (ball driving): The athlete had to drive the ball when the illuminated sensor gave a green light and had to stop when the illuminated sensor gave a red light.

Exc. 5 (transfer—reception): The athlete had to exchange shots with the coach, screaming the color of the illuminated sensor at the time.

Exc. 6 (reflexes): Two athletes stood in front of each other with three illuminated sensors between them. The task was who would first touch the illuminated sensor.

Exc. 7 (abs/strengthening): The athlete had to perform a plank by supporting the body either on the palms or on the elbows and had to pass the hands alternately over the illuminated sensors.

Exc. 8 (strengthening): The athlete had to jump/side jump when the illuminated sensor gave a green light and stay still when the illuminated sensor gave a red light.

Athletes aged 10–12 followed the exercises according to their position: goalkeeper—exercises 1, 2, 3, 7, 8; defenders—exercises 4, 5, 7, 8; midfielders—exercises 4, 5, 6, 7; forwards—all exercises, but mainly 1, 3, 4, 7, 8. Athletes aged 13–15 years old performed exercises 1, 4, 5, 6.

### 2.7. Statistical Analysis of Data

Data are presented as mean ± SD. Data were analyzed using IBM SPSS Statistics v.27.0.1.0. The Shapiro–Wilk Test for normality was used. The normality test determined the method for statistical analysis. Paired *t*-test and Wilcoxon Ranks test were used to examine the mean differences between the two measurements within groups, while Mann–Whitney test and independent *t*-test were used to examine the mean differences (post-measurement—pre-measurement) between the two groups as well as to investigate between-groups differences in pre-test measurements. For some variables, ANCOVA analysis was performed to overcome the fact of initial differences. Moreover, Pearson and Spearman correlation analysis was used to study the association between age, experience, RTs, and RFD values. Finally, linear and multiple regression analyses were performed to add information for the relation between the captured measurements. Two-tailed *p*-values < 0.05 were considered statistically significant.

## 3. Results

**Figure 2:**
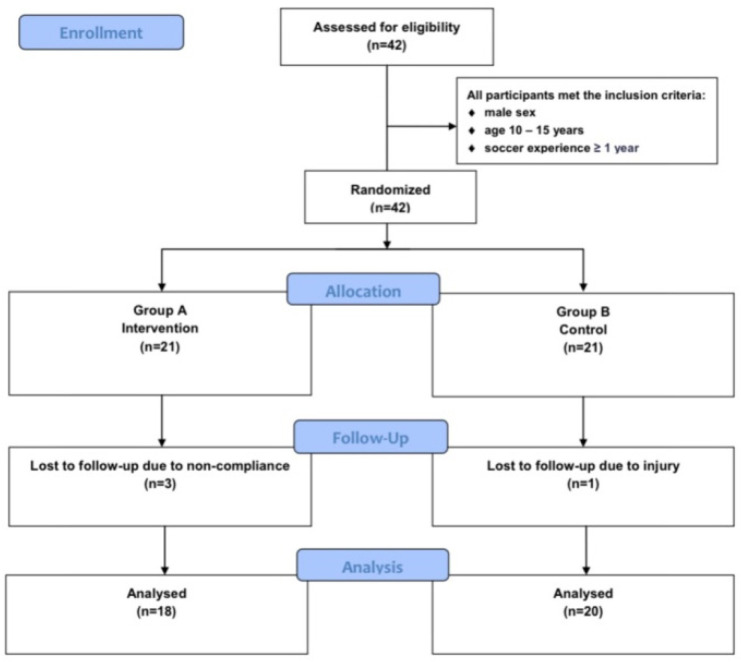
The CONSORT diagram of the study design.

During the 6-month study, four athletes (three from group A and one from B) dropped out of the study due to either injury or non-compliance (Figure 2). Generally, all athletes were very punctual and dedicated to the training program, and any absence was compensated with the valuable help of the coaches to fulfill the appropriate training hours. The mean age of the 38 athletes participating in the study was 12.24 ± 1.76 years old, and their mean soccer experience was 3.32 ± 2.32 years. All 38 athletes fulfilled at least 85% of all exercise training sessions and were analyzed. The clinical and anthropometric characteristics of the athletes at baseline and after 6 months are presented in Table 1. There was no statistically significant difference between the two groups’ clinical and anthropometric data. Although there was a statistically significant difference in the years they played soccer, there was no statistically significant difference in the physical fitness tests at baseline between the two groups, which indicates the homogeneity of the two groups. 

Pre-test measurements were inspected to investigate the initial differences between the two groups. The only variables that showed significant differences were VF (U = 67, *p* < 0.001), pen-to-point dominant hand (U = 50, *p* = 0.003), and pen-to-point non-dominant hand (U = 35, *p* < 0.001). For these measurements, the differences between the two measurements and their statistical significance were examined using ANCOVA analysis. For the required ANCOVA analyses, the covariate was the pre-test measurement in each case, and the dependent variable was the post-test measurement. 

After 6 months, Group A showed statistically significant improvement in SRT by 11.76% (*p* = 0.002, Table 2), repeated sprint by 13.36% (*p* < 0.001, Table 3), and pen-to-point cognitive function test by 71.62% (*p* < 0.001) for the dominant hand and 72.51% (*p* < 0.001) for the non-dominant hand (Table 4). Although VF RT decreased by 8.96%, this was not statistically significant (*p* = 0.384). At the end of the study, Group B showed lower percentages of improvement. The repeated sprint increased by 0.66% (*p* < 0.001) and the pen-to-point cognitive test increased by 64.08% (*p* < 0.001) for the dominant hand and by 58.28% (*p* = 0.002) for the non-dominant hand (Table 5). The Mann–Whitney and independent *t*-test showed statistically significant differences between the two groups for most of the physical fitness measurements, except for the hand grip test (Table 3). In addition, ANCOVA analysis showed significant between-groups differences for VF. 

The RT results assessed using the VR technology are shown in Table 6. Both monocular and binocular isopters were normal for all participants. There was no statistically significant difference either in the within-group change or between the two groups. 

### 3.1. Correlations

Pearson and Spearman correlation analysis (Table 7) was performed and showed significant correlations between age and soccer experience, between age, RT and RFD, and between soccer experience, VF test, and CRT in all athletes. Correlation analysis suggested that reaction time (SRT, CRT) was negatively associated with age; the older (and more experienced) athletes presented lower (quicker) reaction times for both measurements. Age—reaction time correlation seemed reasonable based on the gradual cognitive development of adolescents. In addition, experience was found to have positive effect on VF reaction time and CRT. The latter results also showed a significant relationship between the performance of complex tasks and the experience gained via years of soccer training and playing. Finally, soccer experience affects reaction time, as indicated by the correlation coefficients and the existing literature. Thus, athletes with more years of soccer experience tend to have lower reaction time.

### 3.2. Regression Analysis

Linear and multiple regression analysis were performed on the results of the second measurements taken at the end of the 6-month study to investigate the predictive nature of the measurements regarding FITLIGHT SRT.

#### 3.2.1. Linear Regression

The results of linear regression analysis with SRT as the dependent variable in group A are presented in Table 8. The extracted linear regression models indicated significant relations of SRT variable with VR-VR (*p* < 0.001), CRT (*p* < 0.001), RFD (*p* = 0.006), repeated sprint (*p* = 0.014), hand grip left hand (*p* = 0.046), hand grip right hand (*p* = 0.032), and STR (*p* = 0.012). 

The results showed that the aforementioned variables presented significant prediction capability of STR value. Based on these results, improvements in VR-RT and CRT have a proportional impact on SRT, as well as improvements on the physical fitness tests.

#### 3.2.2. Multiple Regression

Multiple regression analysis (Table 9) was also performed for the second measurement taken at the end of the 6-month study in group A. The model that presented statistically significant results was the one that included all the variables as predictors (F(12, 5) = 13.199, *p* < 0.005, R^2^ = 0.9). From the coefficients, we observed that mainly VR-RT (*t* = 3.393, *p* = 0.019) added to the prediction of SRT. Thus, changes in VR-RT can contribute to changes in SRT. 

## 4. Discussion 

The objective of our study was to observe the effect of a visual stimuli training program on RT, physical condition, and cognitive function in adolescent soccer players. The results of the present study indicate that a 6-month visual training program did not add any further value to the traditional soccer training program in adolescents in regards to RT, physical fitness, and cognitive function. 

Specifically, our results failed to prove the visual training program’s potential to enhance variables relevant to RT, physical fitness, and cognitive function in adolescent soccer players. Even though within-groups statistical tests (Wilcoxon Rank/Paired *t* tests) provided a positive outcome in favor of the visual training program showing statistically significant improvements in SRT, CRT, and cognitive function tests in the athletes who followed the program in comparison to the controls, between-groups statistical tests (Mann–Whitney/Independent *t* tests, ANCOVA) did not show statistically significant differences in the progress of the two groups. Thus, we cannot assume that the training program is actually effective, but its potential should be further investigated. 

In total, our results indicated that RT plays an important role in improving athletic performance and that it can be trained by a visual stimuli program. Likewise, studies have shown that sports, which offer a variety of actions, can influence its development positively [13,45,46]. This is relevant in individual sports, such as swimming, sprint running, and badminton [13,47,48]; as well as in team sports, such as soccer or basketball [13,49,50,51], for quicker response and decision making. We have noticed a significant correlation between age and soccer experience and RT in all athletes. Interestingly, both factors were significantly correlated with CRT, which indicates the identification and selection of a response to various stimuli. This result is in agreement with previous studies that identified these associations [13,16,21,26]. CRT was found to be significantly related to attentional capacity and physical fitness in children and adolescents, suggesting the necessity of psychomotor development programs at that age [13]. Likewise, the athletes in our study had similar baseline performance levels and followed the same soccer training program except for the addition of the visual stimuli program in group A. 

Our results showed that CRT values were higher than SRT for both groups due to the complexity of the task. According to the literature, sports vision training using stimuli is based on the idea that improving visual skills through oculomotor exercises results in sports performance improvement [1,35,48]. Indeed, visual stimuli training is a direct training approach of the oculomotor pathway. Physical condition, cognitive function, and attention have an impact on RT. Thus, the combination of physical exercise and the development of cognitive functioning can improve RT in people. The results of the present study support this claim. 

Lastly, RT in VR measurements did not show any statistically significant improvement. This can be partly explained by considering that RT depends on age, cognitive process, and attention [21,22,23,24], which are very important factors in VR performance. The results of the present study showed statistically significant improvements in VR-RT only in our adolescents of Group A, possibly due to the stabilization of neural system development in this age group. We can assume that the RT in VR technology is similar to CRT due to its complexity. Linear regression analysis proved the similar predictive capability of VR-RT and CRT regarding SRT, a result that agrees with our assumption. 

Another very important factor is neuroplasticity, which is the ability of the human brain to change continuously, and this involves dynamic, structural, and behavioral changes within the nervous system. Once someone achieves or reaches optimal patterns of function, the stabilization of the neural system is developed. Stabilization decreases the system’s capacity to adapt but does not eliminate it [52]. It has been found that exercise training affects the nervous system as it improves blood flow and increases the secretion of neurotrophins [53], which are proteins that regulate neural survival, development, function, synaptic function, and synaptic plasticity [54]. These effects improve the synaptic connectivity and promotes neurogenesis, which are believed to play an important role in enhancing neural plasticity and cognitive function. Neuroplasticity is also important for the improvement of physical fitness [53]. Thus, the improvement of physical and function in our athletes can be attributed to neuroplasticity and exercise-induced neural changes [53]. Keeping in mind all of these physiological processes, we can assume that neuroplasticity is the main factor that leads to the improvement in RT, cognitive function, and physical fitness. 

This study has three main limitations. Firstly, the small size of the sample was mainly due to the 6-month duration of the program. Secondly, only males participated in the study, and finally, we only studied soccer players, mainly due to the popularity of this sport in youth. In any case, this research carries out an interesting analysis in which it has linked variables such as RT, cognitive function, and physical condition using visual stimuli training. 

## 5. Conclusions

The results of the present study indicate that better development of attention and concentration (cognitive function), as well as physical fitness, could improve RT, leading to an improvement in athletic performance, especially during the developmental ages. The within-group improvements were not supported by between-groups significant differences in the progress of the two groups of adolescent athletes. Thus, the potential of a visual stimuli training program should be further investigated. The present study also showed the importance of the quick assessment of RT in the field, which can be achieved using VR technology. Thus, VR technology can be a helpful tool in the hands of coaches and health professionals in the future.

## Figures and Tables

**Table 1:** Clinical and anthropometric characteristics of the athletes.

Clinical Characteristics	Mean Value ± SD		Range (Minimum—Maximum Value)		Independent *t*-Test Report
	Group A	Group B	Group A	Group B	
**Age (years)**	12.72 ± 1.81	11.80 ± 1.64	10–15	10–15	*t* = −1.648, *p* = 0.108
**Height (m)**	1.602 ± 0.112	1.52 ± 0.136	1.41–1.79	1.3–1.8	*t* = −2.065, *p* = 0.066
**Weight (kg)**	52.03 ± 16.24	47.75 ± 14.31	31–90	24–72	*t* = −0.864, *p* = 0.393
**BMI**	19.91 ± 4.34	20.39 ± 4.12	15.5–33.5	13.36–28.4	*t* = −0.354, *p* = 0.726
**Soccer Experience (years)**	5.43 ± 1.74	1.59 ± 0.71	3–8	1–3	*t* = −7.735, *p* < 0.001

BMI: Body Mass Index.

**Table 2:** Simple and complex reaction time measurements at baseline and the end of the 6-month study using FITLIGHT Trainer in both groups.

Reaction Time (s)—Fitlight Trainer	Mean Value ± SD				Statistical Tests Report		Independent *t*-Test Report
	Group A		Group B		Group A	Group B	A vs. B
	Pre	Post	Pre	Post	Wilcoxon Ranks	Wilcoxon Ranks	
**SRT**	0.85 ± 0.15	0.75 ± 0.18	0.84 ± 0.2	0.82 ± 0.14	Z = −3.049, *p* = 0.002	Z = −0.187 *p* = 0.852	*t* = 1.675 *p* = 0.103
**CRT**	0.97 ± 0.13	0.92 ± 0.17	1.08 ± 0.19	1.02 ± 0.13	Z = −2.330, *p* = 0.020	Z = −1.288 *p* = 0.198	*t* = −0.065 *p* = 0.949

SRT: simple reaction time, CRT: complex reaction time, SD: standard deviation, Pre: pre-intervention, Post: post-intervention.

**Table 3:** Physical fitness measurements at baseline and the end of the 6-month study.

Physical Fitness Measures	Mean Value ± SD				Statistical Tests Report (Paired *t*-Test/Wilcoxon Ranks)		Mann–Whitney/ Independen *t*-Test Report
	Group A		Group B		Group A	Group B	A vs. B
	Pre	Post	Pre	Post			
**RFD (Kg/s)**	10.37± 6.03	20.29 ± 13.86	14.57 ± 8.79	19.72 ± 17.23	Z = −3.114 *p* = 0.002	Z = −0.952 *p* = 0.341	U = 115.5 *p* = 0.059
**(R) Hand grip (Kg)**	16.02 ± 8.62	27.69 ± 11.71	12.33 ± 6.11	23.30 ± 9.41	Z = −4.107 *p* < 0.001	Z= −3.920 *p* < 0.001	*t* = −0.536 *p* = 0.595
**(L) Hand grip (Kg)**	14.63 ± 8.94	26.00 ± 10.67	12.56 ± 6.41	24.16 ± 10.01	Z = −4.107 *p* < 0.001	t = −12.521 *p* < 0.001	*t* = 0.194 *p* = 0.847
**Sit-and-reach flexibility (cm)**	4.06 ± 5.79	−3.72 ± 5.63	0.47 ± 5.87	−2.00 ± 6.63	Z = −3.725 *p* < 0.001	Z = −2.279 *p* = 0.023	*t* = 3.978 *p* < 0.001
**Repeated sprint (s)**	21.19 ± 1.17	24.02 ± 2.64	22.78 ± 1.51	22.63 ± 1.44	Z = −3.409 *p* < 0.001	Z = −3.923 *p* < 0.001	U = 36 *p* < 0.001

RFD: rate of force development, (R) hand grip: right hand hand grip, (L) hand grip: left hand hand grip, SD: standard deviation, Pre: pre-intervention, Post: post-intervention.

**Table 4:** Figure drawing test results at baseline and the end of the 6-month study.

Cognitive Test—Figure Drawing	Mean Value ± SD				Statistical tests Report (Paired *t*-Test/Wilcoxon Ranks)		Mann–Whitney/ Independent *t*-Test Report
	Group A		Group B		Group A	Group B	A vs. B
	Pre	Post	Pre	Post			
**Performance (s)—Dominant Hand**	30.86 ± 11.46	38.92 ± 9.83	32.58 ± 15.83	44.68 ± 19.32	Z = −2.638 *p* = 0.008	Z = −2.438 *p* = 0.015	*t* = 0.993 *p* = 0.328
**Pen Lifts—Dominant Hand**	0.75 ± 1.07	0.56 ± 0.71	1.30 ± 1.86	0.79 ± 0.28	t = 0.382 *p* = 0.708	t = 0.362 *p* = 0.722	U = −175 *p* = 0.880
**Performance (s)—Non-Dominant Hand**	25.17 ± 9.09	32.37 ± 9.97	27.68 ± 13.06	33.86 ± 14.71	*t* = −3.106 *p* = 0.007	Z = −1.539 *p* = 0.124	*t* = 0.062 *p* = 0.951
**Pen Lifts—Non-Dominant Hand**	0.63 ± 0.96	0.72 ± 0.90	0.60 ± 1.46	0.40 ± 0.75	*t* = −0.382 *p* = 0.708	*t* = 0.308, *p* = 0.762	U = 163 *p* = 0.578

SD: standard deviation, Pre: pre-intervention, Post: post-intervention.

**Table 5:** Pen-to-point test results at baseline and the end of the 6-month study.

Cognitive Test—Pen to Point (s)	Mean Value ± SD				Statistical Tests Report		ANCOVA Analysis Report
	Group A		Group B		Group A	Group B	A vs. B
	Pre	Post	Pre	Post	Wilcoxon Ranks	Wilcoxon Ranks	
**Dominant Hand**	89.47± 26.69	25.39 ± 6.80	65.89± 27.94	23.67 ± 7.09	Z = −3.351 *p* < 0.001	Z = −3.527 *p* < 0.001	F = 0.542 *p* = 0.467
**Non-Dominant Hand**	98.50 ± 5.60	27.08 ± 7.56	61.58± 30.73	25.69 ± 9.00	Z = −3.516 *p* < 0.001	Z = −3.101 *p* = 0.002	F = 0.196 *p* = 0.661

SD: standard deviation, Pre: pre-intervention, Post: post-intervention.

**Table 6:** Reaction time measurements at baseline and the end of the 6-month study using VR equipment.

Visual Field—Reaction Time (s)	Mean Value ± SD				Statistical Tests Report		ANCOVA Analysis Report
	Group A		Group B		Group A	Group B	A vs. B
	Pre	Post	Pre	Post	Paired *t*-Test	Wilcoxon Ranks	
**VF**	0.67 ± 0.32	0.61 ± 0.31	1.06 ± 0.39	0.88 ± 0.32	*t* = 0.893 *p* = 0.384	Z = 1.717 *p* = 0.086	F = 4.124, *p* = 0.05

VF: visual field test, SD: standard deviation, Pre: pre-intervention, Post: post-intervention.

**Table 7:** Correlation table.

Correlations	Pearson/Spearman Coefficient and *p*-Value	
	Pre	Post
**Age** **—** **SRT**	rho = −0.489, *p* = 0.002	rho = −0.579, *p* < 0.001
**Age** **—** **CRT**	r = −0.360, *p* = 0.026	r = −0.558, *p* < 0.001
**Age** **—** **RFD**	rho = 0.489, *p* = 0.002	rho = 0.571, *p* < 0.001
**Age** **—** **VF**	Not statistically significant	rho = −0.406, *p* = 0.011
**Soccer Experience** **—** **VF**	rho = −0.416, *p* = 0.020	rho = −0.456, *p* = 0.010
**Soccer Experience** **—** **CRT**	r = −0.425, *p* = 0.017	r = −0.469, *p* = 0.008
**Age** **—** **Soccer Experience**	r = 0.445, *p* = 0.012	

SRT: simple reaction time, CRT: complex reaction time, RFD: rate of force development, VF: visual field test, Pre: pre-intervention, Post: post-intervention. r refers to Pearson’s correlation coefficient and rho to Spearman’s correlation coefficient.

**Table 8:** Linear regression analysis—Group A.

Variables	Group A
**VR-RT**	F(1, 16) = 25.070, *p* < 0.001, R^2^ = 0.610
**CRT (** **FITLIGHT** **)**	F(1, 16) = 79.789, *p* < 0.001, R^2^ = 0.833
**DomFigure**	F(1,16) = 0.256, *p* = 0.620, R^2^ = 0.016
**NonDomFig**	F(1,16) = 1.195, *p* = 0.291, R^2^ = 0.069
**DomPen**	F(1,16) = 0.028, *p* = 0.869, R^2^ = 0.002
**NonDomPen**	F(1,16) = 0.131, *p* = 0.722, R^2^ = 0.008
**RFD**	F(1,16) = 9.808, *p* = 0.006, R^2^ = 0.380
**Repeated Sprint**	F(1,16) = 7.693, *p* = 0.014, R^2^ = 0.325
**HandGripL**	F(1,16) = 4.659, *p* = 0.046, R^2^ = 0.226
**HandGripR**	F(1,16) = 5.516, *p* = 0.032, R^2^ = 0.256
**SRT**	F(1,16) = 8.096, *p* = 0.012, R^2^ = 0.336

VR-RT: virtual reality–reaction time (visual field test), CRT (FITLIGHT): complex reaction time using FITLIGHT, DomFigure: figure drawing test for dominant hand, NonDomFig: figure drawing test for non-dominant hand, DomPen: pen-to-point for dominant hand, NonDomPen: pen-to-point for non-dominant hand), RFD: rate of force development, HandGripL: hand grip for left hand, HandGripR: hand grip for right hand, SRT: sit-and-reach flexibility test.

**Table 9:** Multiple regression analysis—Group A: Coefficients of the variables participating in the development of the multiple regression model. VR-RT has the most significant predictive role in this model.

Variables	Group A
**VR-RT**	*t* = 3.393, *p* = 0.019
**CRT (Fitlight)**	*t* = 1.776, *p* = 0.136
**DomFigure**	*t* = 0.473, *p* = 0.656
**NonDomFig**	*t* = −0.768, *p* = 0.477
**DomPen**	*t* = 0.796, *p* = 0.462
**NonDomPen**	*t* = −0.125, *p* = 0.905
**RFD**	*t* = −0.018, *p* = 0.987
**Repeated Sprint**	*t* = 1.088, *p* = 0.326
**HandGripL**	*t* = 1.784, *p* = 0.134
**HandGripR**	*t* = −1.017, *p* = 0.356
**SRT**	*t* = −0.913, *p* = 0.403

## Data Availability

The data of this study are uploaded on Clinicaltrials.gov and are available on request to the corresponding author.
